# Phylogenetic inference from homologous sequence data: minimum topological assumption, strict mutational compatibility consensus tree as the ultimate solution

**DOI:** 10.1186/1745-6150-1-5

**Published:** 2006-02-15

**Authors:** Srdan V Stankov

**Affiliations:** 1 Pasteur Institute, Hajduk Veljkova 1, Novi Sad, Serbia and Montenegro

## Abstract

**Background:**

For the purposes of phylogenetic inference from molecular data sets many different methods are currently offered as alternatives for researchers in phylogenetic systematics. The vast majority of these methods are based on specific topological assumptions relating to the resultant genealogical tree. Each of these has been shown to perform effectively in special conditions and for specific data sets while yielding less reliable results in other instances. Moreover, the majority of the methods include information from homoplastic characters in spite of a universally accepted agreement in their ineffectiveness for phylogenetic inference, which may often lead to inaccuracy and inconsistency. As an alternative to such methods, a strict mutational compatibility consensus tree building method as a universally applicable and reliable method is reported.

**Results:**

The analysis of a data set from a previously published experimental phylogeny demonstrates the accuracy of the strict mutational compatibility consensus tree building method and illustrates its potential for obtaining unambiguous and precise results with full resolution.

**Conclusion:**

The universal applicability of a simplified compatibility method in its algorithmic form for phylogenetic inference is described. Firstly, dismissal of topological assumptions creates a general potential for agreement of inferred with true phylogeny. Second, exclusion of irregular characters from analysis repeatably enables construction of consistent phylogeny. Third, a direct calculation of bootstrap proportion values for individual nodes of the resulting tree is possible rather than their empirical estimation. Finally, guidance is given for empirical assessment of the sample size necessary for full genealogical resolution and significant bootstrap proportions.

**Reviewers:**

This article was reviewed by Yuri I. Wolf (nominated by Eugene Koonin), Arcady Mushegian and Martijn Huynen.

## Open peer review

Reviewed by Yuri I. Wolf (nominated by Eugene Koonin), Arcady Mushegian and Martijn Huynen.

For the full reviews, please go to the Reviewers' comments section.

## Background

Genealogical reconstruction, meaning comprehension of the total scope of ancestor-descendant relationships for a given set of individuals within a population or between different species, has reached its unprecedented resolution with introduction of modern sequencing techniques [1-3]. The general strategy of current character-based methods for an appropriate tree reconstruction from a homologous sequence set relies in the first place on an assumed tree for a given set of taxa. Next, this tree is fitted by various optimality criteria to the partition of character states of an individual character on the taxa, the procedure is repeated for each character and finally a tree is used which best fits all of the characters taken together [1,4]. But, is there a guarantee that all possible tree topologies are considered in the first place? If not, there is also no guarantee that the tree chosen will be the one that absolutely fits to the data. Looking at the topological assumptions made by the current phylogenetic methods, three of them are commonplace in most cases:

a) Analysed taxa are placed exclusively at the terminal nodes of a tree,

b) Each node is labelled by exactly one taxon (either one from the analysed set or one representing a missing ancestor), and

c) The tree is strictly a bifurcating one.

However, there could be no justification for any one of these assumptions. In order to find a tree which fits most to the data, all these assumptions must be rejected, since each of them unnecessarily restricts the set of possible solutions to a particular set of possible topologies. Moreover, instead of first assuming a tree and then finding its fitness to the data, one should start from the data, keeping initial assumptions at a minimum and then let the data create the tree themselves without any further inference from the analyst.

## Results

**Figure 1:**
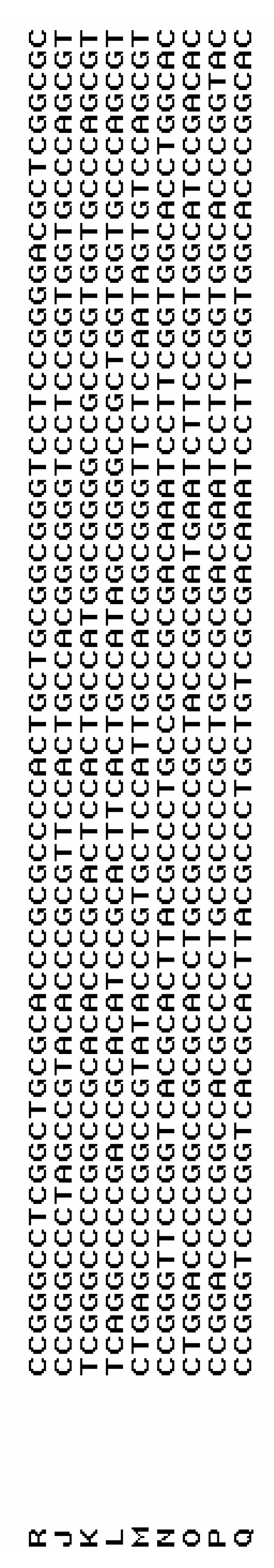
**Input sequence set in a form of alignment**. Alignment of 9 sequences representing phage T7 isolates described in the study of experimental phylogeny of Hillis et al. [6]. Sequences comprise 63 informative sites of a 1091 bp segment of phage T7 genome as presented in Table 1 of Li et al. [5].

**Figure 2:**
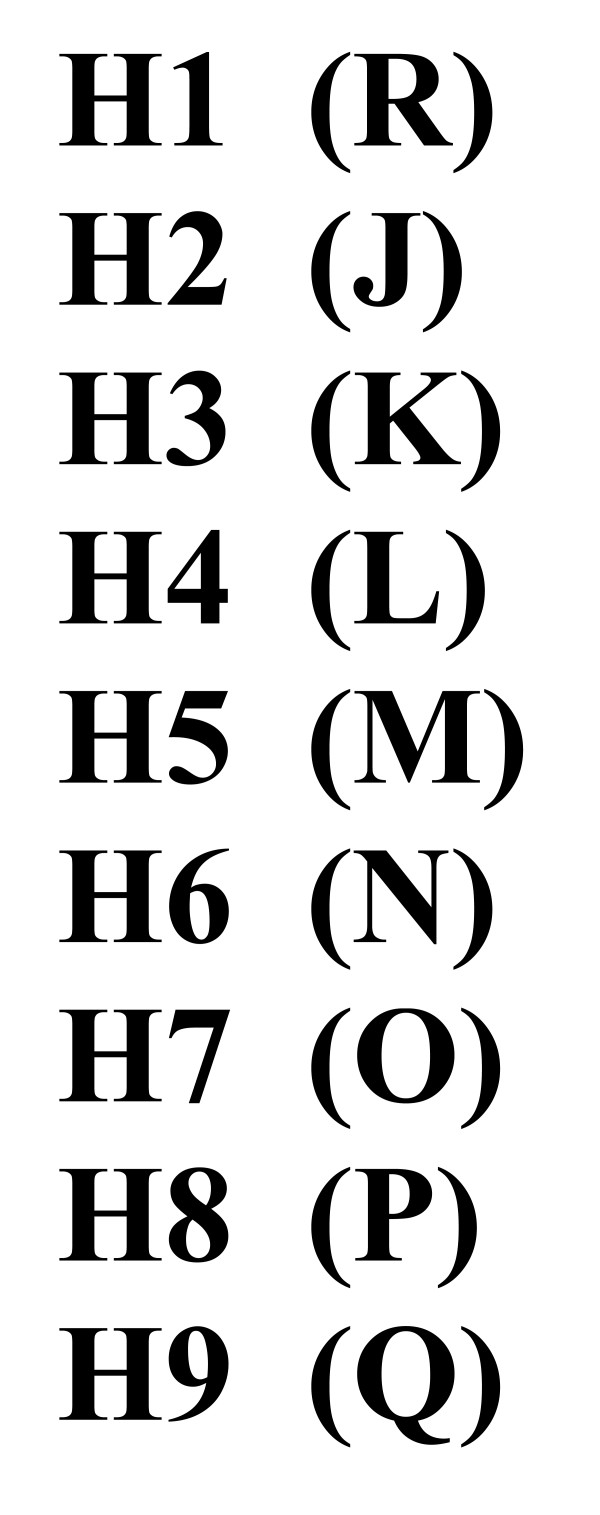
**List of haplotypes**. Ordinary numbers of haplotypes (H) correspond to elements in the input file. Here, each element corresponds to a unique haplotype different from any other one in the list.

**Figure 3:**
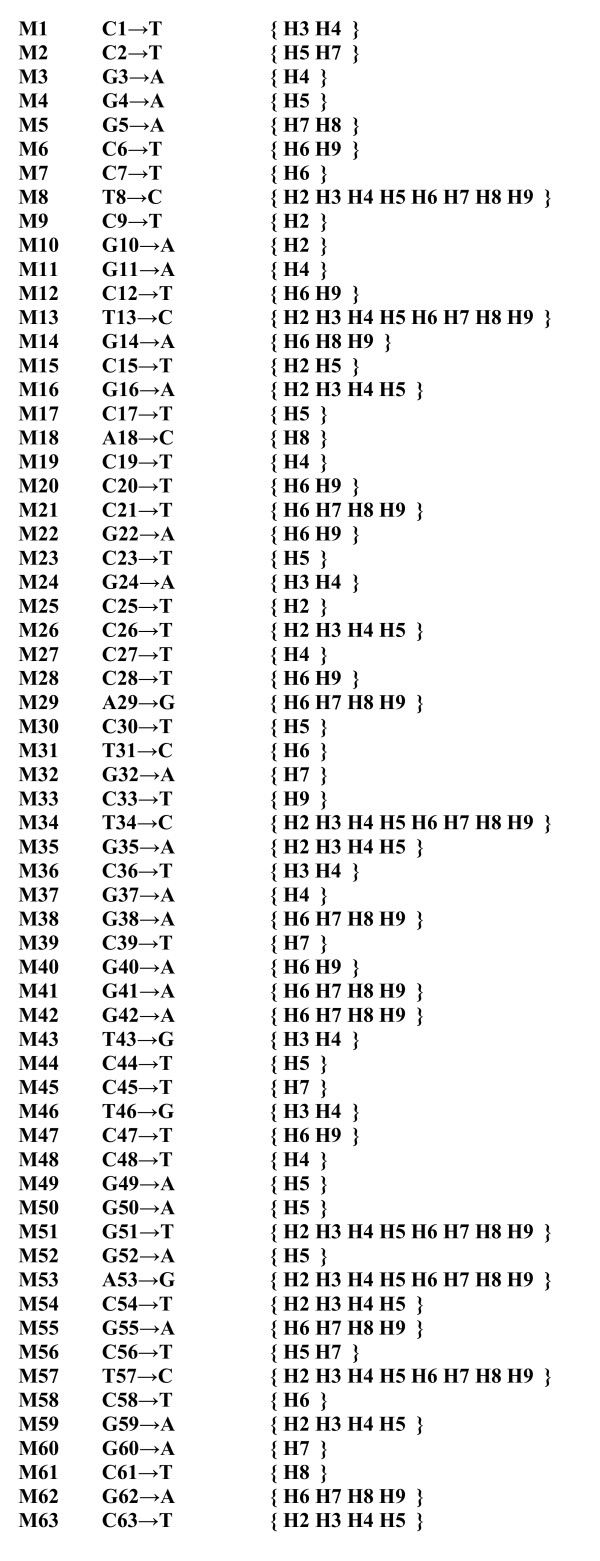
**Marker list**. Each observed marker (M) is represented by the initial base present in the original sequence, base position and finally the derived base. Haplotypes (H) given in parentheses have the derived base of the respective marker.

**Figure 4:**
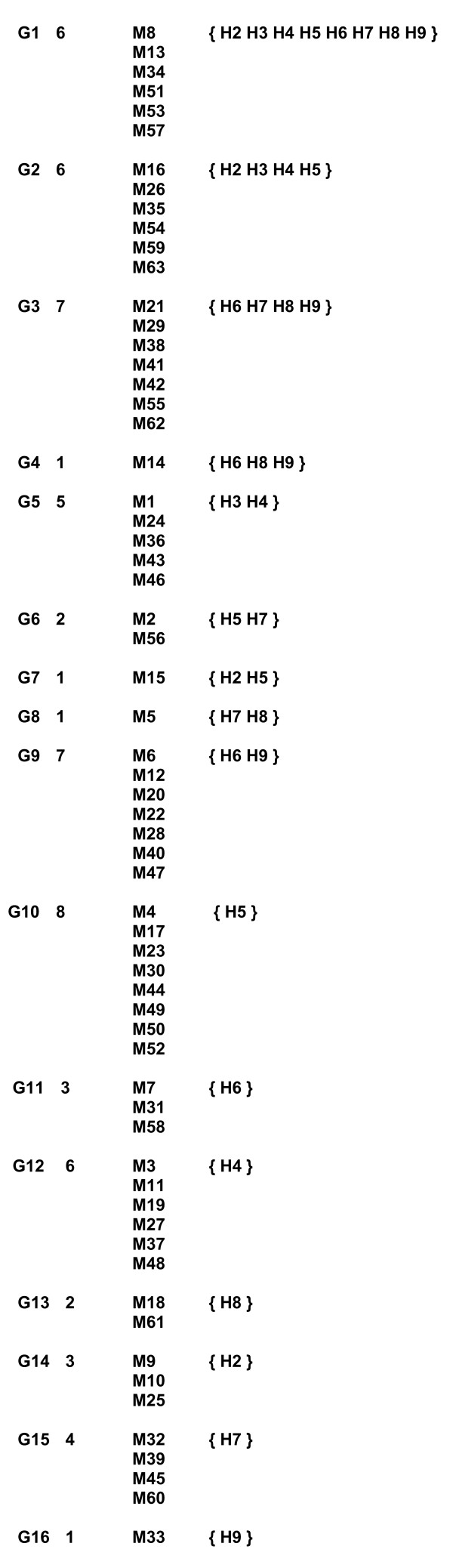
**List of equivalent marker groups**. Equivalent marker groups (G) comprise all markers (M) related to identical haplotype (H) sets given in parentheses. Numbers between designations for groups and markers denote numbers of markers in each group.

**Figure 5:**
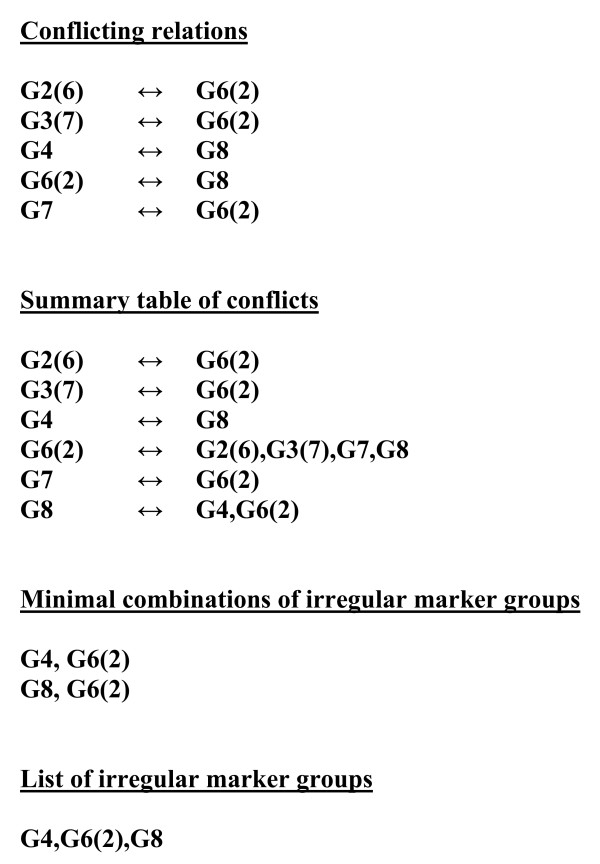
**Conflicting relations and the list of irregular markers**. Different marker groups with haplotype sets forming partial intersections are considered as conflicting (↔) to each other. In the summary table of conflicts all conflicting relations are summarized and presented so that on the left side of each relation a conflicting group is presented alone while on the right side are given all groups conflicting to the latter. The minimal combination(s) of marker groups is chosen which, when removed from the summary table removes all the conflicting relations in the table. All markers included in this minimal combination(s) are designated as irregular markers.

**Figure 6:**
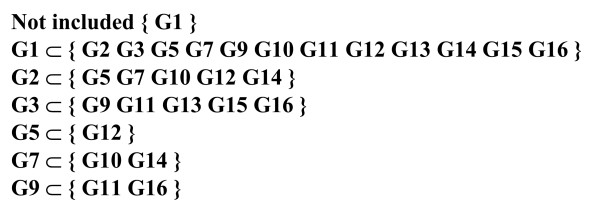
**Total inclusion list**. Designations of marker groups (G) relate here to the corresponding haplotype sets as shown in Figure 4. Each haplotype set included completely in the corresponding set to the left of the inclusion symbol (⊂) is given in parentheses.

**Figure 7:**
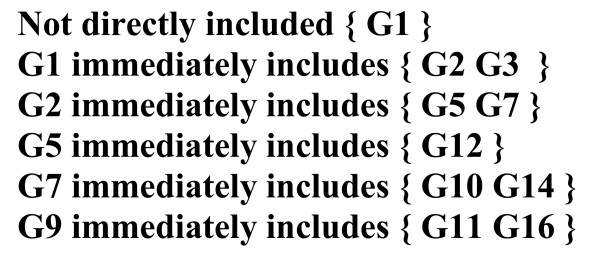
**Immediate inclusion list**. Designations of equivalent marker groups (G) relate here to the corresponding haplotype sets as shown in Figure 4. Each haplotype set given in parentheses is included completely in the corresponding set to the left of the inclusion symbol (⊂), and at the same time not included in any smaller set.

**Figure 8:**
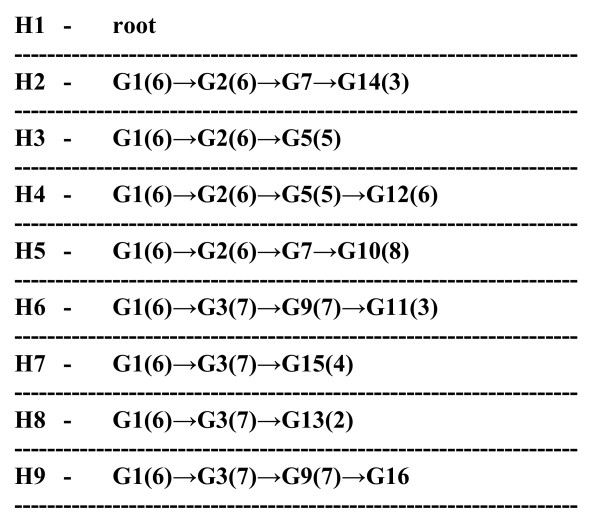
**List of haplotype paths**. Each haplotype (H) is associated with a unique path formed exclusively from regular, mutually compatible marker groups. Paths are represented by chains of marker groups (G), haplotype sets of which all include the respective haplotype. Each set in a chain completely includes its right neighbor. Numbers in brackets denote numbers of equivalent markers in respective groups. Groups without numbers in brackets are singleton groups.

**Figure 9:**
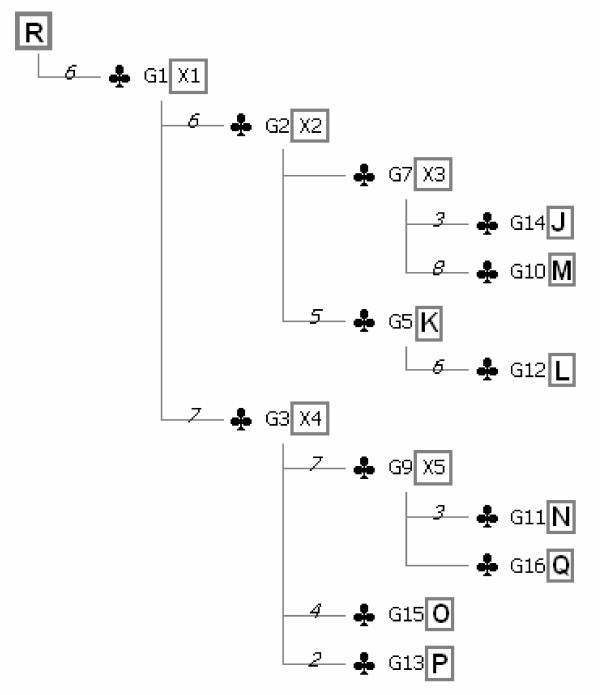
**Strict mutational compatibility consensus tree for phage T7 isolates**. Rooted regular genealogical tree is shown for elements listed in Figure 2. The root (R) is placed on the upper left of the diagram. Each node is represented by a club symbol. Designations of taxa are given in rectangles next to the marker groups (G) at ends of respective regular haplotype paths. Hidden ancestors (X) at internal nodes are shown with ordinary numbers according to their order of appearance on the tree. Numbers at horizontal lines leading to club symbols denote numbers of markers (g values) for neighboring marker groups to the right. Horizontal lines without a number relate to singleton groups.

**Figure 10:**
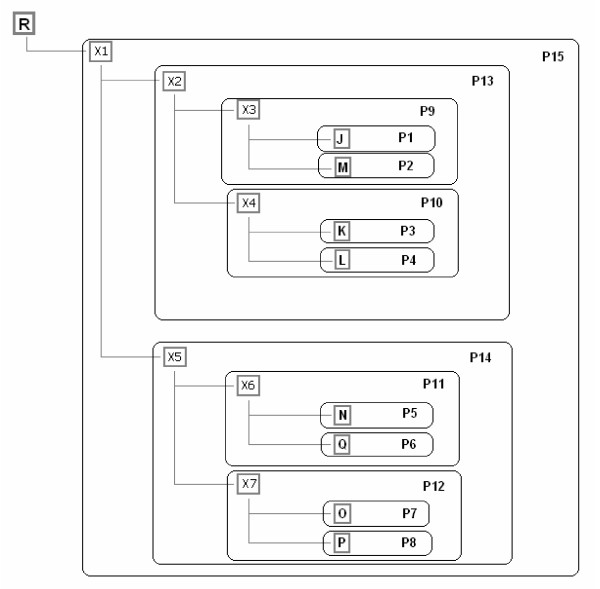
**Plan of experimental phylogeny for phage T7 isolates**. Plan of experimental phylogeny for phage T7 isolates from work of Hillis et al. [16]. The root (R) is placed on the upper left of the diagram. Designations of taxa are given in grey bold rectangles. Hidden ancestors (X) at internal nodes are shown with ordinary numbers according to their order of appearance on the tree. Planned (P) monophyletic groups are shown in black rectangles with designations P1 to P15.

**Figure 11:**
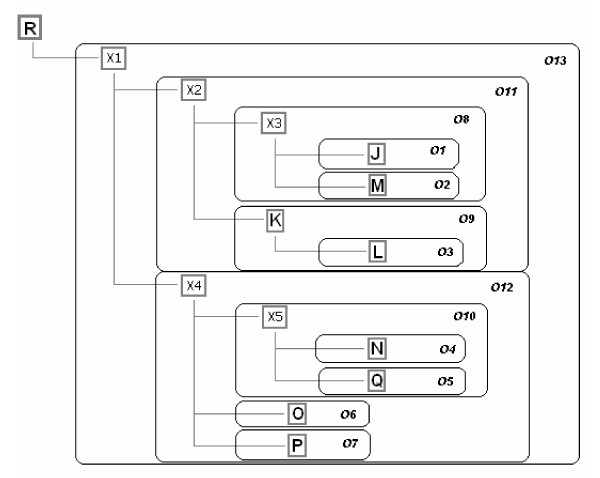
**Observed monophyletic groups on the strict mutational compatibility consensus tree for phage T7 isolates**. Rooted regular genealogical tree is shown for elements listed in Figure 2. The root (R) is placed on the upper left of the diagram. Designations of taxa are given in grey bold rectangles. Hidden ancestors (X) at internal nodes are shown with ordinary numbers according to their order of appearance on the tree. Observed (O) monophyletic groups are shown in black rectangles with designations O1 to O13.

Results of the analysis by the Potomak algorithm of a sequence set (Table 1 in [5]) of T7 phage experimental phylogeny [6] at its consecutive steps are shown in Figures 1, 2, 3, 4, 5, 6, 7, 8, 9. The accuracy of the resulting tree topology was judged by the proportion of observed monophyletic groups of known elements which have counterparts in the experimental plan that are identical by their composition. Figure 10 shows 15 planned monophyletic groups, while Figure 11 shows 13 observed groups, each of which corresponds to one planned group. Hence, the method yielded completely accurate topology. Groups P3 and P12 were not retrieved on the resulting tree indicating incomplete branching resolution because of relatively small sequence length taken into account. With regard to the statistical assessment of the topological precision, there are 10 out of 13 nodes in total (except the root) with significant support, with insignificant values obtained for G7, G13 and G16.

**Table 1:** Theoretical bootstrap support values for a node of a SMCC tree. Bootstrap support values (BS) are given as percentages for various sizes of marker groups (g) and various sequence length (m).

**m ↓ g →**	**1**	**2**	**3**	**4**	**5**
10	65.13 %	89.26 %	97.17 %	99.39 %	99.90 %
100	63.40 %	86.74 %	95.24 %	98.31 %	99.41 %
1000	63.23 %	86.49 %	95.04 %	98.18 %	99.33 %
10000	63.21 %	86.47 %	95.02 %	98.17 %	99.33 %
100000	63.21 %	86.47 %	95.02 %	98.17 %	99.33 %

## Discussion

Parsimony and compatibility are often regarded as very similar methods, hence their properties and utility are generally considered almost equal [4]. The basic difference however, which is overlooked or neglected is the different treatment of homoplastic characters. Whilst parsimony takes into account all characters equally, compatibility makes differences between regular and homoplastic characters and excludes the latter from primary phylogenetic analysis. As a result of a compatibility method, there is always a unique tree, truly reflecting the evolutionary relationships between characters and consequently between analysed elements, whilst results of parsimony analysis are often ambiguous, being represented by numerous equally parsimonious trees.

"If each site in a set of sequences has changed only once in the evolution of a group, then the newly-arisen base will be shared by all species descended from the lineage in which the change occurred. If this were the case at all sites, then the sets of species having the new bases would be either perfectly nested or disjoint, never overlapping unless one set of species was included in the other. It would be possible to erect a tree on which we could explain the evolution of the group with only a single change at each site. This can be done by inspection of the sets of species defined at each varying site. If some of these sets of species overlap without being nested, then there is conflict between the information provided by different sites. Most of the interesting issues in phylogeny reconstruction are in how to resolve these conflicts." [4]

Strategies for resolving homoplasy as revealed by incompatible characters could be divided into two basic categories: a) adaptation of a tree to the full scope of the data, so that the apparent homoplasy on the tree is minimal, and b) recognition of incompatible characters and their exclusion from the phylogenetic analysis [7]. Both approaches regard homoplasy as disturbing, adverse phenomenon. Proponents of the first approach argue that exclusion of homoplastic characters discards information which is still informative regardless of its imperfection. Such a statement is very much alike to a statement that listening to a noise mixed with a pure melody contributes to the artistic value of the melody. As stated by Page and Holmes [8], "homoplasy is a poor indicator of evolutionary relationships, because similarity does not reflect shared ancestry." Since incompatible characters are irrelevant and add no apparent benefit whilst imposing a substantial obstacle to phylogenetic inference, their exclusion from at least primary genealogical analysis is fully justified. Once a regular tree has been formed, irregular markers could be used for elucidation of the natural history of reversions, parallelisms and also recombinations. Thus, reverse mutations form paraphyletic groups rooted by the marker which has subsequently reverted, whilst parallel mutations form polyphyletic groups, each monophyletic subgroup of which is being rooted by the same marker. Recombinations usually cause multiple parallelisms or multiple reversions within a single element, whereby markers placed between these are always younger, formed after the moment of recombination. Hence, most of recombinations can also be detected on the resulting tree. A detailed consideration of such events, however, falls beyond the scope of this paper and will be described elsewhere.

Absence of topological assumptions allows any tree topology to be deduced from the data, including polytomies of unlimited size. Besides the strategy of tree construction directly from the integrity of the (compatible) data at hand, the presented method differs from the classical compatibility methods in the creation of groups of equivalent markers to simplify the analysis. While the accuracy of the SMCC tree in relation to the analysed data is ensured by the presented Potomak algorithm, a tree resulting from clique analysis, even in case that the maximal consensus clique is taken may not be true simply because of topological restrictions imposed by individual character state trees used for formation of the final tree. Character state changes are here analogous to binary factors of directed non-binary cladistic characters. Multiple markers at the same site must comprise mutually disjoint haplotype sets because one haplotype can have only one character state at one site. Consequently, a star phylogeny for the respective character state tree is in principle assumed. However, eventual irregular markers at this site are subsequently discarded. SMCC tree is most closely related to a tree obtained by clique analysis of binary data when maximal consensus clique is used for tree construction. Thus, when the T7 data set as given in Figure 1 is analysed by program Clique, package Phylip version 3.64 [9] the only topological differences of the output tree in relation to the SMCC tree in Figure 9 are placements of R and K according to the unjustified assumption a) above at separate branches with zero character state changes for each of them. Since their length equals zero, they are artificial, i.e. non-existing in reality. Otherwise, designations of character changes along each branch entirely correspond to the ones on the SMCC tree (Additional file 1). The consensus of mutually compatible character state changes should not be confused with consensus trees, namely trees chosen by certain criteria from a forest previously obtained by current tree-to-data adapting methods. So it is the compatibility consensus, not a consensus tree which is referred to here.

### Accuracy of the method

Comparing the SMCC tree topology for the experimental T7 phylogeny (Figure 9) with the phylogeny of T7 as planned and conducted by Hillis et al. [6], one should note that there are three points of disagreement: first, in the SMCC tree there is no common ancestor for K and L after X2; there is also no common ancestor for O and P after X4; and third, L originates directly from K instead from their common ancestor. How could these discrepancies be explained? First, one should make an accurate distinction between haplotype genealogy and reproductive genealogy. What is directly revealed by the genetic character analysis is haplotype genealogy. Conversely, reproductive genealogy could be directly revealed only by the direct observation of reproductive history of organisms, as in this case, which was undertaken throughout the experiment with T7 phage. In the absence of such a possibility, haplotype genealogy is used for indirect inference of the former. For the total agreement of two genealogies however, enough genetic markers for discerning each step in the corresponding reproductive history of analysed elements must be observed. Here, a relatively small segment of T7 genome yielded 13 regular groups in total. One could expect that almost certainly the above mentioned discrepancies would be solved with the inclusion of additional genome segments in analysis. However, it could have also been the case that throughout the whole genome only these 13 groups were recorded. In that case it would have been impossible to reveal the true reproductive history by genetic analysis.

### Mutation model assumed

In reality, a genealogical method should take into account the deterministic nature of the DNA replication process as well as the exceptional nature of mutational occurrence. This forms precisely the basis of the first step of any phylogenetic analysis – establishment of identity-by-descent as a basic feature of homologous characters. An important mutation model which has proven its value in population genetics and which fully respects the exceptional nature of mutational occurrence is the 'infinite sites model' [10,11]. It is a universally applicable model even when data comprises some fast-changing sites for the following reason. Homology as an obligate pre-requisite implies identity of a substantial proportion of characters across all taxa analysed. For these sites, the mutation rate equals zero. Since there is no discontinuous transition from sites with zero mutation rate to a high mutation rate, there is always a class of sites with a minimal mutation rate fully conforming to the infinite sites model, and this class of sites is precisely the one most valuable for phylogenetic inference. But what happens when numerous sites are identified with high mutation rates? Fast-changing sites usually imply the appearance of many irregular markers in the data set, leaving paucity of regular ones for construction of the SMCC tree. As a consequence, relatively low resolution is obtained. In such unpleasant situations we just have to accept the fact that the available data are simply not suitable and do not allow for proper phylogenetic analysis. Such an example is shown in Additional files 2 and 3. The aligned bacterial ribosomal RNA sequences are nearly randomized, with typical pairwise similarities below 50%, yielding paucity of regular markers and poorly resolved SMCC tree with insignificant BS values. With such sets, any phylogenetic analysis reduces to a pure guesswork – a procedure that could be equally well conducted even without looking at sequence data, thus saving the efforts and costs of sampling and DNA sequencing.

### Dependence of resolution on the sample size taken

Low resolution means that there are many analysed elements placed in groups of two or more at individual nodes. One may refer to this kind of resolution as 'apparent resolution', which differs from the branching resolution (i.e. resolution between hidden ancestors) described in the results section, however one should bear in mind that both depend on much the same factor. How should this problem be managed? "If the largest clade contains very few characters, then skepticism concerning the suitability of the data set for promoting a valid estimate of evolutionary history is justified. The ability of character compatibility analysis to fail in this manner should be considered an advantage: One is less likely to propose a tree based on insubstantial evidence." [12] The Potomak algorithm offers a clue for the choice of the minimal sequence length sufficient for full genealogical resolution given the number of elements taken for analysis when a pilot study revealing the occurrence and frequency of regular marker groups has already been conducted. In a tree with full resolution, each node must be labelled by not more than one element. The number of regular marker groups must in that case be greater than the number of elements analysed. Assuming that marker group frequency for an unresolved tree increases linearly with sequence length for a fixed number of analysed elements (N), and taking into account the analysed DNA length (l) and the number of groups (n, less than N) obtained in a pilot study, the minimal length needed would be (N/n) l. However, if the objective were a tree with all its nodes being significantly supported, a different criterion would have to be used. For fully resolved trees, one could not expect that the number of marker groups increases further. Instead, with increasing sequence length g values rise linearly for each group, with highest increase rate for largest groups and lowest rates for smallest groups as a consequence of different evolution rates for different branches. One could in principle assume a linear distribution of g values for a particular sample size yielding full resolution. For a precise estimate of group sizes for any DNA length one should have data from multiple sampling examples so that the function of the slope change is elucidated. However, for the most optimistic estimate one could rely on one sampling example on assumption that the regression line slope does not change significantly. In our example, taking into account eleven g values in decreasing order for non-singleton groups (for more precise calculation), one gets a linear regression function y = 8.04 - 0.57 x. Here, for x = 13 (corresponding to the last node) the estimated value for the group size is 0.6. In order that the estimated g value for the last group reaches 3 (sufficient for significant BS), estimated group sizes should rise for 2.4, with the size of the largest group reaching 10.4. Taking the size of the largest group as most precisely correlating to the sequence length sampled, for achieving the above mentioned condition one should take the sequence length of (10.4/8) × 1091 = 1417 units.

### Consistency of the method

One should also note that the tree is consistent in a sense that all partitions found with a certain sequence segment are preserved when longer segments which include the first segment are analysed, along with further nested grouping of elements within previously obtained groups. This feature is analogous with taking pictures at increasingly higher resolutions. For example, on a low-resolution picture of a man only head, trunk and extremities may be discernible. At a higher resolution one could observe further details on the head – eyes and nose; on extremities – hands and feet etc., whereby initial division of the body into head, trunk and extremities as noted at low resolution remains preserved. The only case in which this tree consistency might be disturbed is definition of a monophyletic group by a marker group which with a greater sequence length turns out to be irregular. This can happen most often with singleton groups, less frequently with groups with two markers and extremely rarely with g values of 3 or more. A further example of the applicability of the presented algorhitm on natural sequence sets is given in Additional file 4, where the feature of consistency is clearly shown.

No simulations were attempted with the described method because "the conclusions of such studies all too often seem to match pre-existing preferences of the authors. This problem arises because all methods have conditions for which they work well, and other conditions for which they work poorly. It is relatively easy to identify the optimal conditions of a favourite method, and then present simulation results that compare competing methods only at this optimum. Such results are of very limited interest, but the conclusions drawn from such studies often are presented as if they were general." [13]

## Method

### Methodology for reconstruction of a strict mutational compatibility consensus (SMCC) tree without topological assumptions – general principles

Each individual (taxon, evolutionary unit, element) in a given set is represented by a single nucleotide or amino acid sequence. A character state change (point mutation, insertion or deletion), defined as a triad of the ancestral sequence unit, unit position and the derived sequence unit is called a marker. Markers are in principle mutually independent. However, deletions or insertions of two or more consecutive bases are due to their interdependence counted as a single marker. As with other methods, a necessary pre-requisite is the appropriate sequence alignment [14,15].

Phylogenetic reconstruction begins with the designation of the original sequence. Often, this sequence is not known, so one has to choose a sequence which genealogically does not belong to the analysed group (outgroup), but is similar enough so that the ancestral character states are properly designated.

Once established derived state reproduces through next generations, forming a set of haplotypes bearing the derived state (marker's haplotype set, or simply marker set). Further, a second marker formed within an element of this set relates to a set of haplotypes which represents its subset etc. In other words, regular marker set relationships are such that a haplotype set of a marker formed earlier in the course of the genealogical history completely includes one formed later in an element already bearing the derived state of the first marker. Such evolutionary scenario leads to the formation of 'compatible characters'. Relations of compatibility concerning morphologic characters were given due consideration by Hennig [16] and a procedure for testing compatibility of phylogenetic hypotheses under the term "consistency" was developed by Wilson [17]. The specific algorithm for the examination of character compatibility and selection of compatible binary characters was given by Le Quesne [18], generalized later for multistate characters by Estabrook [19,20]. Finally, Meacham and Estabrook [12] highlighted the importance of exclusion of incompatible characters for phylogenetic analysis. A phylogeny created by compatible characters has been called by computer scientists a "perfect phylogeny", one formed exclusively by nested sets of elements such that for any two sets one completely includes or excludes the other [7,21]. In contrast, markers formed by reverse mutations may only partially include smaller sets, while parallel mutations may comprise marker sets which are only partially included by larger sets. Since their relationships to other sets are not regular with respect to perfect phylogeny, such markers are referred to as 'irregular markers'. For proper construction of a regular tree, all irregular markers should first be recognized and excluded from further analysis, so that a strict consensus of compatible markers remains. Finally, forking chains of immediate inclusions of regular marker sets growing from the root form a unique strict mutational compatibility consensus (SMCC) tree. Here, a marker group uniquely labels each node, and haplotypes are located at nodes labelled by the marker group with the smallest haplotype set in which they appear. Looking at a particular node, three possibilities for its resulting haplotype labels may arise. First, concerning the monophyletic group rooted at the node, all mutually exclusive monophyletic subsets of the group rooted in neighboring distal nodes together include all haplotypes of the group. In this case the node is represented by an unknown haplotype, not present in the given data set. Second, the above mentioned subsets taken together include all but one element of the group, meaning that the node is represented by this missing element. Finally, when two or more elements are not included in any of the distal subsets, the node is labelled by all of them at the same time.

### Potomak – algorithm for SMCC tree reconstruction from homologous sequence data with results from an example of experimental phylogeny

For the purpose of clarity, the algorithm for construction of an SMCC tree is here presented along with its results in each step of analysis of a sequence set (Table 1 in [5]) of T7 phage experimental phylogeny [6].

1. Sequences, including the original or outgroup, have to be presented in the form of alignment (Fig. 1),

2. List of haplotypes is formed, where each element is represented by the first one in each group of identical sequences (Fig. 2),

3. Designation of the original haplotype,

4. Marker list is formed on which each mutation in relation to the original sequence is shown as a marker. Each marker on the list relates to the corresponding set of haplotypes bearing the marker, or more precisely its derived character state (Fig. 3),

5. List of groups of equivalent markers (or simply marker groups) is formed by grouping markers related to identical sets of haplotypes. Numbers associated with marker groups denote numbers of markers included in each group (Fig. 4),

6. Those marker groups whose haplotype sets partially include each other are recognized as conflicting groups. All conflicting relations are then summarized and presented in one summary table of conflicts. Then, the minimal combination of markers is chosen which, when removed from the summary table removes all the conflicting relations so that the table disappears (Fig. 5). Markers included in this minimal combination are designated as irregular (homoplastic) markers, while the rest represents the maximal combination of compatible characters. In special cases when there are two or more minimal combinations which lead to removal of the table of conflicts, all characters appearing in any one of them are considered irregular and hence excluded from further analysis. In this case one does not get a strict maximal set of compatible markers, but strict consensus set of compatible markers leading to a unique, unambiguous directed tree.

7. Next, the inclusion list for regular marker groups presents for each group all other groups whose haplotype sets are completely included in its own set (Fig. 6), with initial notification of groups with sets not included in any other set.

8. Immediate inclusion list is formed by choosing for each group only those groups from the previous list whose sets are not included in any other group's smaller set (Fig. 7),

9. Groups with sets not included in any larger set on the latter list are placed in direct connection with the root. Growing of the tree is then continued along forking chains of immediate inclusions to their ends. Each haplotype is then placed with the marker group with the smallest set containing the haplotype (Figure 8). Thus, each node except the root is represented by a unique marker group and a known haplotype at the end of the respective haplotype path or for internal nodes by an unknown haplotype when known haplotypes are absent. Finally, designations of elements corresponding to haplotypes can be shown in place of haplotype designations (Figure 9). Here, each element corresponds to a unique haplotype, but in other sequence sets this is often not the case.

The straightforwardness of this algorithm ensures that there is only one unambiguously constructed genealogical tree for each sequence set analysed. Since the topology of the tree is determined exclusively by relations of regular marker sets directly deduced from the data, the tree construction is devoid of any topological assumptions set in advance to the algorithm itself.

### Assessment of bootstrap support values for a SMCC tree

One of the most widely accepted tests for the degree of confidence in classification of taxa into subtrees (monophyletic groups) on a given tree is the determination of the bootstrap support value [22]. On a SMCC tree, one can for each subtree precisely calculate the theoretical bootstrap support value, namely the value which would be obtained if the number of bootstrap replicates reached infinity. A particular subtree appears in a bootstrap replicate here if at least one site corresponding to a marker defining the root of the group is sampled. Suppose that this node is defined by a marker group of g equivalent markers placed at g different sites. Then, since formation of a replicate is equivalent to m times sampling one site out of m sites in total with replacement, the probability that neither one of g sites would be taken in one drawing is (m-g)/m. Further, the probability that neither one of g sites would be taken in m consecutive drawings (necessary for formation of one bootstrap replicate) is ((m-g)/m)^m^. Conversely, the probability that at least one of g sites would appear in a bootstrap replicate is BS = 1 - ((m-g)/m)^m^, as Felsenstein [22] noted for the case of a compatible data set. This latter probability represents in this way the theoretical bootstrap support value (BS) for a given node. The precise values for BS are given in the Table 1 for ranges of values g from 1 to 5 and m from 10 to 100000.

As is evident from the presented table, a node generated by a group of three or more equivalent markers receives a significant BS value of over 95 % for the sequence length of at least 100000 units.

## Conclusion

In summary, a rooted SMCC tree has the following essential properties:

a) It is devoid of any topological assumption except for strict requirement for a tree-like structure without reticulations,

b) Nodes are determined by regular groups of equivalent markers,

c) Each element is unambiguously placed at an internal node or a tip of the tree,

d) All hidden ancestors at internal nodes are shown, and

e) Numbers of regular markers arising between each two nodes are given at their links, so that one can calculate the number of regular mutations occurring between any two elements on the same path. Therefore the tree could be regarded as a metric one.

Provided the sequence length is greater than a certain critical size, this method yields unique, consistent, fully resolved and well supported genealogical tree for individuals, populations or species with further possibility of determination of nature and positions of irregular mutations.

## Reviewers' comments

### Reviewer's report 1

Yuri I. Wolf, National Center for Biotechnology Information, National Library of Medicine, National Institutes of Health, Bethesda, MD, USA (nominated by Eugene V. Koonin, National Center for Biotechnology Information, National Library of Medicine, National Institutes of Health, Bethesda, MD, USA)

Comments from Eugene V. Koonin:

An attempt to develop an "ultimate" phylogenetic algorithm free of (almost) any assumptions is laudable. However, this paper does not offer any evidence that the algorithm would produce informative results on simulated or real-life data of any complexity (see the review by Yuri Wolf). A thorough revision including a detailed comparison to other algorithms and assessment of performance on various classes of data could change things.

*Author response: The main objective of the paper was to present the underlying principles of the phylogenetic method in its algorithmic form and to point out its main features by analysis of one well known experimental data set. The algorithm allows the sequence data to form a unique result by their own, without any inference from the analyst. So the informativeness of the result is here determined by the nature and extent (sample size) of the sequence data analysed, not by the algorithm itself. The algorithm guarantees accuracy, whereby informativeness inasmuch it relates to the obtained tree resolution depends on the analysed data and not on the algorithm itself, as explained in the Discussion section. So the algorithm cannot be compared with other algorithms that by themselves produce "informativeness" without ensuring accuracy. Assessment of performance on various classes of data is surely needed, but this task might be too extensive for a single paper. Of practical importance here is the fact that the paper offers a tool for obtaining objective results, thus widening the choice of methods available for phylogeneticists without precluding use of any other method as an alternative, if preferred by the investigator*.

Comments from Yuri I. Wolf

The paper by SV Stankov describes a compatibility-based algorithm proclaimed to be the "ultimate solution" of the phylogenetic inference problem. The algorithm involves grouping of haplotypes (unique sequences) according to shared phylogenetic markers (directed mutations, inferred from the ancestral or outgroup sequence) and removing the minimal set of markers that form homoplasies (incompatible group assignments). Mutually compatible groups are assembled into a hierarchical tree-like structure on the strength of inclusion of lower-ranked sets into the higher-ranked ones. The algorithm is free from tree topology constraints and can produce multifurcations and place extant sequences in internal tree nodes.

The full assessment of the algorithm novelty requires a review by a "hardcore" phylogeneticists and cannot be provided by this reviewer.

*Author response: This paper surely needs to be viewed and reviewed by "hardcore" phylogeneticists since it presents the very "hardcore" of phylogenetics*.

The paper does not contain any extensive analysis of the robustness of the reconstructed phylogeny and comparison with existing methods. The reviewer is skeptical about the practical usefulness of strict compatibility reconstruction as complex data would tend to produce star-like trees (where all extant haplotypes are attached to a single ancestral node) due to extensive incompatibility between makers. The paper does not contain any evidence suggesting otherwise.

*Author response: Star-like or any other SMCC tree topology can in no case result from incompatibility between markers, since incompatible markers are removed in advance, hence they cannot influence the tree construction procedure in any way. Besides, one should in principle accept the fact that in reality, genealogy is indeed star-like. Any parent with more than two children forms with them a star-like tree (or a star-like subtree)*.

### Reviewer's report 2

Arcady R. Mushegian, Bioinformatics Center, Stowers Institute for Medical Research, Kansas City MO, USA

Reviewer comments:

1. State clearly what is the difference between this method and other compatibility methods, in particular those that rely on finding cliques.

*Author response: Relevant statements were already included in the manuscript version presented to the reviewer. These are "Besides the strategy of tree construction directly from the integrity of the (compatible) data at hand, the presented method differs from the classical compatibility methods in the creation of groups of equivalent markers to simplify the analysis." (page 4, line 48 – page 5, line 1) and "Since the topology of the tree is determined exclusively by relations of regular marker sets directly deduced from the data, the tree construction is devoid of any topological assumptions set in advance to the algorithm itself." (page 9, line 21 – 24)*.

However, for a specific comparison of the presented method with clique analysis, I inserted a new sentence on page 5, lines 1–5 in the final version:

"While the accuracy of the SMCC tree in relation to the analysed data is ensured by the presented Potomak algorithm, a tree resulting from clique analysis, even in case that the maximal consensus clique is taken may not be true simply because of topological restrictions imposed by individual character state trees used for formation of the final tree."

Reviewer comments:

I would be satisfied with the example showing how clique method is failing to obtaining the same results on the same T7 dataset, and discussion of the difference.

2. All sites in the T7 dataset are two-state sites. What about three and four states – if method becomes more complicated or not applicable, say so.

Author response: Relevant remark is given on page 5, lines 2–6: "Multiple markers at the same site must comprise mutually disjoint haplotype sets because one haplotype can have only one character state at one site. Consequently, a star phylogeny for the respective character state tree is in principle assumed. However, eventual irregular markers at this site are subsequently discarded."

Reviewer comments:

1. Legend to Figure 1: the numbers attached to the references do not match the numbers in the reference list.

*Author response: The mistake was correctly noticed, so I made the appropriate changes in the final version*.

Reviewer comments:

2. In the background section, the emphasis seems to be on three common constraints on the tree topology, which the current method seeks to overcome. The conditions a. and c., however, are not hard to drop – there are methods that do not require them – and I am not sure what condition b. is all about. But, anyway, it is unclear what the rest of the paper has to do with all this. Only a. seems to be relevant to the data that are analyzed, viz. K being the parent of L without such assumption or sister group of L with it, and even this is discussed only as the shortcoming of the method, not as significant difference. I am confused.

*Author response: The incomplete branching resolution (resulting in K being the parent of L) observed in the given example is not a shortcoming of the method itself, but a consequence of insufficient sequence length analyzed (i.e. insufficient sample size). "enough genetic markers for discerning each step in the corresponding reproductive history of analysed elements must be observed. Here, a relatively small segment of T7 genome yielded 13 regular groups in total. One could expect that almost certainly the above mentioned discrepancies would be solved with the inclusion of additional genome segments in analysis. However, it could have also been the case that throughout the whole genome only these 13 groups were recorded. In that case it would have been impossible to reveal the true reproductive history by genetic analysis." (page 5, lines 24–31)*.

Reviewer comments:

Nonetheless, I do not see how the critique of generally assumed a., b. and c. is germane to the paper.

3. Implementation details and complexity analysis of the algorithm would be helpful. Please provide.

*Author response: I intend to present implementation details and complexity analysis of the algorithm as soon as the appropriate program is technically perfected (which is currently not the case), along with a free presentation of the program on a web site*.

Reviewer comments.

Generally, primary papers cited in the manuscript are all from the 1980s; references for the algorithmic work in the 1990s can be found, for example, in Felsenstein's book (Inferring Phylogenies, Sinauer Associates, 2004). Not essential, but would improve reader's understanding of the state of the art.

### Reviewer's report 3

Martijn A. Huynen, Center for Molecular and Biomolecular Informatics Nijmegen Centre for Molecular Life Sciences, Radboud University Nijmegen Medical Centre, Nijmegen, Netherlands

Reviewer comments:

The the best of my knowledge (I am not a character compatibility expert) the approach that is presented is new, the most interesting point being the development of an algorithm that identifies a set of strictly mutationally compatibel mutations in a set of aligned sequence data, and deriving of a tree from that. The latter is in contrast to methods that"test" all possible trees for their compatibility with a set of sequences. Because the algorithm derives the tree, it allows a less strict definition of what a tree should look like, a less strict definition of course leads to more possible trees, and makes explicit testing computationally harder.

Looming behind the article is of course the perennial debate how strict one should be methodologically in deriving trees for sequence data. This manuscript takes a rather extreme viewpoint, not even adressing the debate between heuristic distance/clustering approaches, some of which do allow for multifurcating trees, and character-based approaches, but even within the character-based approaches selecting only a set of markers (positions) that is strictly compatibel with itself. I will not address this discussion here, however I do think that describing including characters that are to some extent homoplastic as adding pure noise to a pure melody does not contribute anything to the discussion. We all would like to use only the perfect, non-homoplastic characters, but in practice we often have little choice: there are just not enough of those.

My first main concern is whether the algorithm is of any practical use to people who regularly make phylogenies from sequence data. One example is shown in which the algorithm is succesfully applied to an experimental phylogeny of T7 sequences that was constructed by Hillis et al. That set of sequences has the advantage that the initial state is know for all sequences. Generally we do not know the initial state of sequences, even if an outgroup is available. Specifically in the sequencing of genomes from species for which we do not have fossil data, or in situations where Horizontal Gene Transfer might have occurred it is often not possible to obtain an outgroup.

The author argues against the usage of simulation data to test his method, because then he might generate those data that would fit his criteria. I would still like to see the method tested on a larger set of sequence data from "real life": e.g. take a set of (aligned) ribosomal RNA sequences, e.g. 50 from the bacteria, and run the method: how many positions are useful?

My second main concern is the following: The algorithm is presented as non-parametric: it chooses the largest set of compatibel mutations, or, at least, it removes the minimal combination of markers that when removed, leave all the other positions compatibel with each other. But what if the dataset contains multiple compatible sets that are (almost) equally large? Furthermore, is the algorithm guaranteed to find the largest compatible set? The second concern actually is related to my wish to see the algorithm tested on a set of biological sequences, to have an example of how many positions there are in the compatibility consensus.

I guess the culprit lies in the remark that "Provided the sequence length is greater than a critical size the method yields unique, consistent, fully resolved and well supported genealogical tree(s, ed.) for individuals" A calculation about the "Dependence of resolution on the sample size taken" gives theoretical values of the required sequence length for a certain set of sequences. Again, how do those numbers play out for biological sequences?

It is common practice to make the code of a published algorithm available. Can the Potomak program be put on a web site?

Technical comment

Homology, as detected by current day, profile-based methods, does not require identity of a substantial proportion characters across all taxa.

In general the method is well described. The author puts the emphasis on the fact that he has a less constrained tree than do other methods, aside from the remark that e.g. a method like Tree Puzzling does also not produce fully resolved trees, I was most intrigued by the algorithm that is used to get at the compatible set of characters, and derive the tree from that.

Summarizing:

I would like to see a major revision that basically addresses the questions above regarding practical usability and the issue of how the algorithm deals with multiple, (almost) equally large sets of compatible markers in the aligned sequences.

I think the paper is of importance in its field, provided the algorithm is shown to be of partical use: i.e. applying it on a reasonably large set of biological sequences there are enough positions in the compatibility consensus set to obtain a phylogeny.

I have no competing interests regarding the publication of this paper.

## Declaration of competing interests

The author(s) declare that they have no competing interests.

## Supplementary Material

Additional File 1 Clique analysis of phage T7 data set. Input file is formed from the alignment in Figure 1 in two steps. First, ancestral states were given "0", while derived states were given "1" designation. Next, derived states for characters 5 and 14 were changed so that both of them got equivalent with characters 2 and 56. With such modified input file and using shown options the program Clique, package Phylip version 3.64 produced a single clique comprising the same 59 regular characters used for construction of the SMCC tree in Figure 9 along with the corresponding compatibility tree (well viewed by WordPad v5.1). Click here for file

Additional File 2 SMCC analysis of a bacterial ribosomal RNA data set (well viewed by WordPad v5.1). Click here for file

Additional File 3 SMCC tree for the data set from Additional file 2 Click here for file

Additional File 4 SMCC analysis of influenza A nucleoprotein gene sequence set Click here for file
