# Supplementary figures and images for: Phylogenetic inference from homologous sequence data: minimum topological assumption, strict mutational compatibility consensus tree as the ultimate solution
Source: Biol Direct. 2006 Feb 15;1:5. doi: 10.1186/1745-6150-1-5 (PMC1409768; doi:10.1186/1745-6150-1-5)

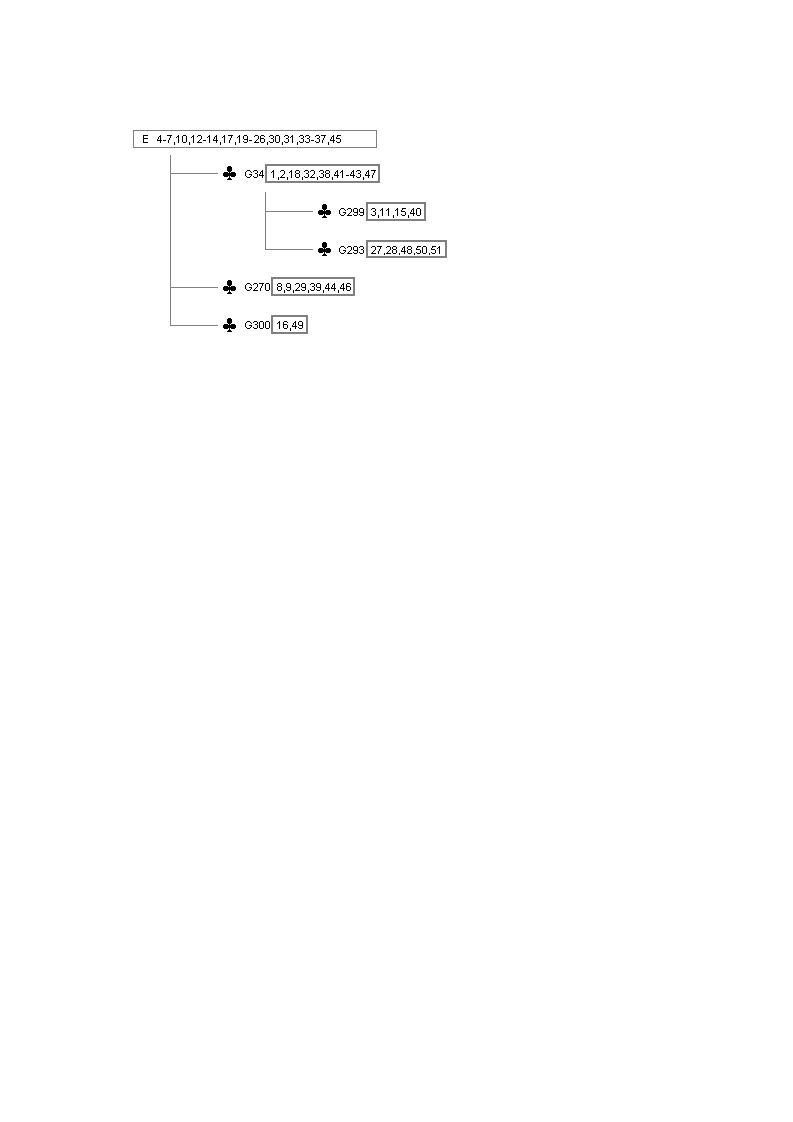

Supplement: Additional File 3 — SMCC tree for the data set from Additional file 2 [file 1745-6150-1-5-S3.doc]
