# Supplementary material for: Phylogenetic inference from homologous sequence data: minimum topological assumption, strict mutational compatibility consensus tree as the ultimate solution
Source: Biol Direct. 2006 Feb 15;1:5. doi: 10.1186/1745-6150-1-5 (PMC1409768; doi:10.1186/1745-6150-1-5)
Supplement: Additional File 4 — SMCC analysis of influenza A nucleoprotein gene sequence set [file 1745-6150-1-5-S4.rtf]

SECTION I 

Input sequence set
Different haplotypes in a collection of 161 isolate of influenza A virus type H3N2 from the territory of Holland in the period from 1989 till 1999 (Voeten JT, Bestebroer TM, Nieuwkoop NJ, Fouchier RA, Osterhaus AD, Rimmelzwaan GF. Antigenic drift in the influenza A virus (H3N2) nucleoprotein and escape from recognition by cytotoxic T lymphocytes. J Virol 2000;74:6800-7.); In the left column are ordinary numbers of elements; in the right column are designations of isolates in the Nucleotide database [http://www.ncbi.nlm.nih.gov/entrez/query.fcgi?db=Nucleotide].
E1	 AF225709
E2	 AF225716
E3	 AF225744
E4	 AF225745
E5	 AF225746
E6	 AF225747
E7	 AF225748
E8	 AF225750
E9	 AF225751
E10	 AF225752
E11	 AF225754
E12	 AF225757
E13	 AF225758
E14	 AF225759
E15	 AF225760
E16	 AF225762
E17	 AF225763
E18	 AF225764
E19	 AF225782
E20	 AF225783
E21	 AF225784
E22	 AF225785
E23	 AF225788
E24	 AF225790
E25	 AF225796
E26	 AF225798
E27	 AF225801
E28	 AF225811
E29	 AF225812
E30	 AF225815
E31	 AF225818
E32	 AF225823
E33	 AF225831
E34	 AF225833
E35	 AF225834
E36	 AF225836
E37	 AF225839
E38	 AF225846
E39	 AF225849
E40	 AF225850
E41	 AF225851
E42	 AF225852
E43	 AF225853
E44	 AF225855
E45	 AF225866
E46	 AF225867


SECTION II	

SMCC analysis for the segment 834 – 947 of the influenza A nucleoprotein gene	


List of haplotypes

H1	(E1,E3,E4,E5,E6,E7,E8,E9,E12,E13,E14,E15,E16,E17,E18,E20,
	E22,E23,E24,E26,E27,E28,E29,E30,E31,E34,E36)
H2	(E2)
H3	(E10)
H4	(E11)
H5	(E19)
H6	(E21)
H7	(E25)
H8	(E32)
H9	(E33,E35,E41)
H10	(E37,E38,E39,E40,E43)
H11	(E42)
H12	(E44,E45,E46)


Marker list

M1 b3->t	{ H3 H9 H11  }
M2 b15->g	{ H5  }
M3 b29->a	{ H8 H9 H11  }
M4 b38->g	{ H8 H9 H11  }
M5 b47->g	{ H7  }
M6 b48->g	{ H2  }
M7 b63->g	{ H12  }
M8 b64->t	{ H3 H4  }
M9 b78->g	{ H11  }
M10 b99->t	{ H10  }
M11 b105->g	{ H6  }


List of equivalent marker groups

G1	1	M1	{ H3 H9 H11  }

G2	2	M3	{ H8 H9 H11  }
		M4

G3	1	M8	{ H3 H4  }

G4	1	M5	{ H7  }

G5	1	M6	{ H2  }

G6	1	M7	{ H12  }

G7	1	M2	{ H5  }

G8	1	M9	{ H11  }

G9	1	M10	{ H10  }

G10	1	M11	{ H6  }


Summary table of conflicts

G1	<->	G2(2),G3
G2(2)	<->	G1
G3	<->	G1


Irregular groups

G1

Total inclusion list

Not included { G2 G3 G4 G5 G6 G7 G9 G10 }
G2 C { G8 }


Immediate inclusion list

Not immediately included { G2 G3 G4 G5 G6 G7 G9 G10 }
G2 immediately includes { G8 }


List of haplotype paths

H1	=	root
------------------------
H2	-	G5
------------------------
H3	-	G3
------------------------
H4	-	G3
------------------------
H5	-	G7
------------------------
H6	-	G10
------------------------
H7	-	G4
------------------------
H8	-	G2(2)
------------------------
H9	-	G2(2)
------------------------
H10	-	G9
------------------------
H11	-	G2(2)->G8
------------------------
H12	-	G6
------------------------


SMCC tree with monophyletic groups


SECTION III	

SMCC analysis for the segment 720 – 947 of the influenza A nucleoprotein gene	


List of haplotypes

H1	(E1,E4,E5,E6,E7,E12,E13,E17)
H2	(E2)
H3	(E3)
H4	(E8)
H5	(E9)
H6	(E10)
H7	(E11)
H8	(E14)
H9	(E15,E16)
H10	(E18,E20,E22,E23,E26,E27,E28,E30)
H11	(E19)
H12	(E21)
H13	(E24,E31)
H14	(E25)
H15	(E29)
H16	(E32)
H17	(E33,E35,E41)
H18	(E34)
H19	(E36)
H20	(E37,E38,E40)
H21	(E39,E43)
H22	(E42)
H23	(E44,E45)
H24	(E46)


Marker list

M1 b3->c	{ H24  }
M2 b18->t	{ H23 H24  }
M3 b24->t	{ H8  }
M4 b30->g	{ H3 H12 H13  }
M5 b42->t	{ H3  }
M6 b42->a	{ H21  }
M7 c59->b	{ H1 H2 H3 H4 H5 H6 H7 H8 H9 H23 H24  }
M8 b60->a	{ H6 H9 H15  }
M9 b73->c	{ H12 H16 H17 H18 H19 H20 H21 H22  }
M10 b75->g	{ H23 H24  }
M11 b87->a	{ H4  }
M12 b93->c	{ H19  }
M13 b96->a	{ H5  }
M14 b105->a	{ H16 H17 H18 H19 H22  }
M15 b117->t	{ H6 H17 H22  }
M16 b129->g	{ H11  }
M17 b143->a	{ H16 H17 H22  }
M18 b152->g	{ H16 H17 H22  }
M19 b161->g	{ H14  }
M20 b162->g	{ H2  }
M21 b177->g	{ H23 H24  }
M22 b178->t	{ H6 H7  }
M23 b192->g	{ H22  }
M24 b213->t	{ H20 H21  }
M25 b219->g	{ H12  }


List of equivalent marker groups

G1	1	M7	{ H1 H2 H3 H4 H5 H6 H7 H8 H9 H23 H24  }

G2	1	M9	{ H12 H16 H17 H18 H19 H20 H21 H22  }

G3	1	M14	{ H16 H17 H18 H19 H22  }

G4	1	M4	{ H3 H12 H13  }

G5	1	M8	{ H6 H9 H15  }

G6	1	M15	{ H6 H17 H22  }

G7	2	M17	{ H16 H17 H22  }
		M18

G8	3	M2	{ H23 H24  }
		M10
		M21

G9	1	M22	{ H6 H7  }

G10	1	M24	{ H20 H21  }

G11	1	M12	{ H19  }

G12	1	M13	{ H5  }

G13	1	M6	{ H21  }

G14	1	M3	{ H8  }

G15	1	M16	{ H11  }

G16	1	M1	{ H24  }

G17	1	M19	{ H14  }

G18	1	M20	{ H2  }

G19	1	M5	{ H3  }

G20	1	M23	{ H22  }

G21	1	M11	{ H4  }

G22	1	M25	{ H12  }


Summary table of conflicts

G1	<->	G4,G5,G6
G2	<->	G4,G6
G3	<->	G6
G4	<->	G1,G2
G5	<->	G1,G6,G9
G6	<->	G1,G2,G3,G5,G7(2),G9
G7(2)	<->	G6
G9	<->	G5,G6


Irregular groups

G4,G5,G6


Total inclusion list

Not included { G1 G2 G15 G17 }
G1 C { G8 G9 G12 G14 G16 G18 G19 G21 }
G2 C { G3 G7 G10 G11 G13 G20 G22 }
G3 C { G7 G11 G20 }
G7 C { G20 }
G8 C { G16 }
G10 C { G13 }


Immediate inclusion list

Not immediately included { G1 G2 G15 G17 }
G1 immediately includes { G8 G9 G12 G14 G18 G19 G21 }
G2 immediately includes { G3 G10 G22 }
G3 immediately includes { G7 G11 }
G7 immediately includes { G20 }
G8 immediately includes { G16 }
G10 immediately includes { G13 }


List of haplotype paths

H1	-	G1
------------------------
H2	-	G1->G18
------------------------
H3	-	G1->G19
------------------------
H4	-	G1->G21
------------------------
H5	-	G1->G12
------------------------
H6	-	G1->G9
------------------------
H7	-	G1->G9
------------------------
H8	-	G1->G14
------------------------
H9	-	G1
------------------------
H10	=	root
------------------------
H11	-	G15
------------------------
H12	-	G2->G22
------------------------
H13	-	
------------------------
H14	-	G17
------------------------
H15	-	
------------------------
H16	-	G2->G3->G7(2)
------------------------
H17	-	G2->G3->G7(2)
------------------------
H18	-	G2->G3
------------------------
H19	-	G2->G3->G11
------------------------
H20	-	G2->G10
------------------------
H21	-	G2->G10->G13
------------------------
H22	-	G2->G3->G7(2)->G20
------------------------
H23	-	G1->G8(3)
------------------------
H24	-	G1->G8(3)->G16
------------------------


SMCC tree with monophyletic groups


SECTION IV	

SMCC analysis for the segment 720 – 1175 of the influenza A nucleoprotein gene	


List of haplotypes

H1	(E1)
H2	(E2)
H3	(E3)
H4	(E4)
H5	(E5)
H6	(E6)
H7	(E7)
H8	(E8)
H9	(E9)
H10	(E10)
H11	(E11)
H12	(E12)
H13	(E13)
H14	(E14)
H15	(E15)
H16	(E16)
H17	(E17)
H18	(E18)
H19	(E19)
H20	(E20)
H21	(E21)
H22	(E22)
H23	(E23)
H24	(E24)
H25	(E25)
H26	(E26)
H27	(E27)
H28	(E28)
H29	(E29)
H30	(E30)
H31	(E31)
H32	(E32)
H33	(E33)
H34	(E34)
H35	(E35)
H36	(E36)
H37	(E37)
H38	(E38)
H39	(E39)
H40	(E40)
H41	(E41)
H42	(E42)
H43	(E43)
H44	(E44)
H45	(E45)
H46	(E46)


Marker list

M1 b3->c	{ H46  }
M2 b18->t	{ H44 H45 H46  }
M3 b24->t	{ H14  }
M4 b30->g	{ H3 H21 H24 H31  }
M5 b42->t	{ H3  }
M6 b42->a	{ H39 H43  }
M7 c59->b	{ H1 H2 H3 H4 H5 H6 H7 H8 H9 H10 H11 H12 H13 H14 H15 H16 H17 H44 H45 H46  }
M8 b60->a	{ H10 H15 H16 H29  }
M9 b73->c	{ H21 H32 H33 H34 H35 H36 H37 H38 H39 H40 H41 H42 H43  }
M10 b75->g	{ H44 H45 H46  }
M11 b87->a	{ H8  }
M12 b93->c	{ H36  }
M13 b96->a	{ H9  }
M14 b105->a	{ H32 H33 H34 H35 H36 H41 H42  }
M15 b117->t	{ H10 H33 H35 H41 H42  }
M16 b129->g	{ H19  }
M17 b143->a	{ H32 H33 H35 H41 H42  }
M18 b152->g	{ H32 H33 H35 H41 H42  }
M19 b161->g	{ H25  }
M20 b162->g	{ H2  }
M21 b177->g	{ H44 H45 H46  }
M22 b178->t	{ H10 H11  }
M23 b192->g	{ H42  }
M24 b213->t	{ H37 H38 H39 H40 H43  }
M25 b219->g	{ H21  }
M26 b237->a	{ H4  }
M27 b237->t	{ H44 H45 H46  }
M28 b265->t	{ H27  }
M29 b270->a	{ H41 H42  }
M30 c288->b	{ H1 H2 H3 H4 H5 H6 H7 H8 H9 H10 H11 H12 H13 H14 H15 H16 H17 H21 H32 H33 H34 H35 H36 H37 H38 H39 H40 H41 H42 H43 H44 H45 H46  }
M31 b304->c	{ H6  }
M32 a306->b	{ H1 H2 H4 H6 H7 H8 H9 H11 H12 H13 H15 H16 H17  }
M33 b312->a	{ H15 H34 H36  }
M34 b315->g	{ H42 H46  }
M35 b324->a	{ H19 H26 H28  }
M36 b324->t	{ H23  }
M37 b331->t	{ H22 H30  }
M38 b339->g	{ H13  }
M39 g342->b	{ H1 H2 H3 H4 H5 H6 H7 H8 H9 H10 H11 H12 H13 H14 H15 H16 H17 H21 H32 H33 H34 H35 H36 H37 H38 H39 H40 H41 H42 H43 H44 H45 H46  }
M40 b345->t	{ H34 H36  }
M41 b348->a	{ H30 H34 H36  }
M42 b351->a	{ H36  }
M43 b363->c	{ H27  }
M44 b365->c	{ H14 H17  }
M45 b366->g	{ H10  }
M46 b384->g	{ H10  }
M47 b405->a	{ H28  }
M48 a407->b	{ H1 H2 H3 H4 H5 H6 H7 H8 H9 H10 H11 H12 H13 H14 H15 H16 H17 H31 H44 H45 H46  }
M49 b411->g	{ H44 H45 H46  }
M50 b423->g	{ H39  }
M51 b426->a	{ H35  }
M52 b429->g	{ H39 H43  }
M53 a433->b	{ H1 H2 H3 H4 H5 H6 H7 H8 H9 H10 H11 H12 H13 H14 H15 H16 H17 H44 H45 H46  }
M54 b434->a	{ H19 H20 H26 H28  }
M55 b435->a	{ H38  }
M56 b444->a	{ H37 H38 H41  }
M57 b449->c	{ H45  }
M58 b450->a	{ H7  }
M59 b453->a	{ H12  }


List of equivalent marker groups

G1	2	M30	{ H1 H2 H3 H4 H5 H6 H7 H8 H9 H10 H11 H12 H13 H14 H15 
H16 H17 H21 H32 H33 H34 H35 H36 H37 H38 H39 H40 H41 
H42 H43 H44 H45 H46  }
		M39


G2	1	M48	{ H1 H2 H3 H4 H5 H6 H7 H8 H9 H10 H11 H12 H13 H14 H15
 H16 H17 H31 H44 H45 H46  }

G3	2	M7	{ H1 H2 H3 H4 H5 H6 H7 H8 H9 H10 H11 H12 H13 H14 H15
 H16 H17 H44 H45 H46  }
		M53


G4	1	M32	{ H1 H2 H4 H6 H7 H8 H9 H11 H12 H13 H15 H16 H17  }

G5	1	M9	{ H21 H32 H33 H34 H35 H36 H37 H38 H39 H40 H41 H42 H43  }

G6	1	M14	{ H32 H33 H34 H35 H36 H41 H42  }

G7	1	M15	{ H10 H33 H35 H41 H42  }

G8	2	M17	{ H32 H33 H35 H41 H42  }
		M18


G9	1	M24	{ H37 H38 H39 H40 H43  }

G10	1	M4	{ H3 H21 H24 H31  }

G11	1	M8	{ H10 H15 H16 H29  }

G12	1	M54	{ H19 H20 H26 H28  }

G13	1	M35	{ H19 H26 H28  }

G14	1	M41	{ H30 H34 H36  }

G15	5	M2	{ H44 H45 H46  }
		M10
		M21
		M27
		M49


G16	1	M33	{ H15 H34 H36  }

G17	1	M56	{ H37 H38 H41  }

G18	1	M37	{ H22 H30  }

G19	1	M40	{ H34 H36  }

G20	2	M6	{ H39 H43  }
		M52


G21	1	M44	{ H14 H17  }

G22	1	M29	{ H41 H42  }

G23	1	M34	{ H42 H46  }

G24	1	M22	{ H10 H11  }

G25	1	M13	{ H9  }

G26	1	M1	{ H46  }

G27	1	M31	{ H6  }

G28	1	M5	{ H3  }

G29	1	M16	{ H19  }

G30	1	M3	{ H14  }

G31	1	M19	{ H25  }

G32	1	M36	{ H23  }

G33	1	M20	{ H2  }

G34	1	M38	{ H13  }

G35	1	M11	{ H8  }

G36	1	M23	{ H42  }

G37	2	M12	{ H36  }
		M42


G38	2	M45	{ H10  }
		M46


G39	1	M47	{ H28  }

G40	1	M25	{ H21  }

G41	1	M50	{ H39  }

G42	1	M51	{ H35  }

G43	1	M26	{ H4  }

G44	1	M55	{ H38  }

G45	2	M28	{ H27  }
		M43

G46	1	M57	{ H45  }

G47	1	M58	{ H7  }

G48	1	M59	{ H12  }


Summary table of conflicts

G1(2)	<->	G2,G10,G11,G14
G2	<->	G1(2),G7,G10,G11,G16,G23
G3(2)	<->	G7,G10,G11,G16,G23
G4	<->	G11,G16,G21,G24
G5	<->	G7,G10,G14,G16,G23
G6	<->	G7,G14,G16,G17,G23
G7	<->	G2,G3(2),G5,G6,G8(2),G11,G17,G23,G24
G8(2)	<->	G7,G17,G23
G9	<->	G17
G10	<->	G1(2),G2,G3(2),G5
G11	<->	G1(2),G2,G3(2),G4,G7,G16,G24
G14	<->	G1(2),G5,G6,G16,G18
G15(5)	<->	G23
G16	<->	G2,G3(2),G4,G5,G6,G11,G14
G17	<->	G6,G7,G8(2),G9,G22
G18	<->	G14
G21	<->	G4
G22	<->	G17,G23
G23	<->	G2,G3(2),G5,G6,G7,G8(2),G15(5),G22
G24	<->	G4,G7,G11


Irregular groups

G2,G4,G7,G10,G11,G14,G16,G17,G23


Total inclusion list

Not included { G1 G12 G18 G31 G32 G45 }
G1 C { G3 G5 G6 G8 G9 G15 G19 G20 G21 G22 G24 G25 G26 G27
 G28 G30 G33 G34 G35 G36 G37 G38 G40 G41 G42 G43 G44 G46 G47 G48 }
G3 C { G15 G21 G24 G25 G26 G27 G28 G30 G33 G34 G35 G38 G43 G46 G47 G48 }
G5 C { G6 G8 G9 G19 G20 G22 G36 G37 G40 G41 G42 G44 }
G6 C { G8 G19 G22 G36 G37 G42 }
G8 C { G22 G36 G42 }
G9 C { G20 G41 G44 }
G12 C { G13 G29 G39 }
G13 C { G29 G39 }
G15 C { G26 G46 }
G19 C { G37 }
G20 C { G41 }
G21 C { G30 }
G22 C { G36 }
G24 C { G38 }


Immediate inclusion list

Not immediately included { G1 G12 G18 G31 G32 G45 }
G1 immediately includes { G3 G5 }
G3 immediately includes { G15 G21 G24 G28 }
G5 immediately includes { G6 G9 G40 }
G6 immediately includes { G8 G19 }
G8 immediately includes { G22 G42 }
G9 immediately includes { G20 G44 }
G12 immediately includes { G13 }
G13 immediately includes { G29 G39 }
G15 immediately includes { G26 G46 }
G19 immediately includes { G37 }
G20 immediately includes { G41 }
G21 immediately includes { G30 }
G22 immediately includes { G36 }
G24 immediately includes { G38 }


List of haplotype paths

H1	-	G1(2)->G3(2)
------------------------
H2	-	G1(2)->G3(2)->G33
------------------------
H3	-	G1(2)->G3(2)->G28
------------------------
H4	-	G1(2)->G3(2)->G43
------------------------
H5	-	G1(2)->G3(2)
------------------------
H6	-	G1(2)->G3(2)->G27
------------------------
H7	-	G1(2)->G3(2)->G47
------------------------
H8	-	G1(2)->G3(2)->G35
------------------------
H9	-	G1(2)->G3(2)->G25
------------------------
H10	-	G1(2)->G3(2)->G24->G38(2)
------------------------
H11	-	G1(2)->G3(2)->G24
------------------------
H12	-	G1(2)->G3(2)->G48
------------------------
H13	-	G1(2)->G3(2)->G34
------------------------
H14	-	G1(2)->G3(2)->G21->G30
------------------------
H15	-	G1(2)->G3(2)
------------------------
H16	-	G1(2)->G3(2)
------------------------
H17	-	G1(2)->G3(2)->G21
------------------------
H18	=	root
------------------------
H19	-	G12->G13->G29
------------------------
H20	-	G12
------------------------
H21	-	G1(2)->G5->G40
------------------------
H22	-	G18
------------------------
H23	-	G32
------------------------
H24	-
------------------------
H25	-	G31
------------------------
H26	-	G12->G13
------------------------
H27	-	G45(2)
------------------------
H28	-	G12->G13->G39
------------------------
H29	-
------------------------
H30	-	G18
------------------------
H31	-
------------------------
H32	-	G1(2)->G5->G6->G8(2)
------------------------
H33	-	G1(2)->G5->G6->G8(2)
------------------------
H34	-	G1(2)->G5->G6->G19
------------------------
H35	-	G1(2)->G5->G6->G8(2)->G42
------------------------
H36	-	G1(2)->G5->G6->G19->G37(2)
------------------------
H37	-	G1(2)->G5->G9
------------------------
H38	-	G1(2)->G5->G9->G44
------------------------
H39	-	G1(2)->G5->G9->G20(2)->G41
------------------------
H40	-	G1(2)->G5->G9
------------------------
H41	-	G1(2)->G5->G6->G8(2)->G22
------------------------
H42	-	G1(2)->G5->G6->G8(2)->G22->G36
------------------------
H43	-	G1(2)->G5->G9->G20(2)
------------------------
H44	-	G1(2)->G3(2)->G15(5)
------------------------
H45	-	G1(2)->G3(2)->G15(5)->G46
------------------------
H46	-	G1(2)->G3(2)->G15(5)->G26
------------------------


SMCC tree with monophyletic groups
